# Tailoring the Optimized Fermentation Conditions of SCOBY-Based Membranes and Milk Kefir Grains to Promote Various Functional Properties

**DOI:** 10.3390/foods11193107

**Published:** 2022-10-06

**Authors:** Marina Pihurov, Bogdan Păcularu-Burada, Mihaela Cotârleț, Gabriela Elena Bahrim

**Affiliations:** Faculty of Food Science and Engineering, Dunarea de Jos University of Galati, Domneasca Street No. 111, 800201 Galati, Romania

**Keywords:** SCOBY-based membranes, milk kefir grains, co-fermentation, bioprocess optimization, bioactives

## Abstract

Kombucha culture (named SCOBY-Symbiotic Culture of Bacteria and Yeasts) and milk kefir grains represent multiple consortia of wild microorganisms that include lactic acid bacteria, acetic acid bacteria and yeasts with valuable functional properties. Their fermentative potential provides a wide range of derivate metabiotics (prebiotics, probiotics, postbiotics and paraprobiotics) with valuable in vitro and in vivo benefits. This study targeted the evaluation of the functionality of a co-culture of SCOBY-based membranes and milk kefir grains, used as freeze-dried starter cultures, for the fermentation of a newly formulated medium based on black tea infusion, supplemented with bovine colostrum and sugar, in order to produce bioactive compounds with functional properties. The design and optimization of the biotechnological process were achieved by using the Plackett–Burman experimental design (six factorial points, three center points) and the response surface methodology and central composite design (three factorial points, six axial points and two center points in axial) tools. The statistical analysis and the mathematical modelling of the responses such as the pH, titratable acidity, antioxidant activity and antimicrobial activity (against *Bacillus subtilis*, *Escherichia coli*, *Staphylococcus aureus* and *Aspergillus niger*) were investigated. Further, the composition of organic acids, polyphenols and flavonoids of the fermented product obtained under the optimized fermentation conditions was also analyzed. The fermentation of the medium containing 6.27% (*w*/*v*) bovine colostrum powder, 1.64% (*w*/*v*) black tea, 7.5% (*w*/*w*) sugar, pH 6.7, with an inoculum based of 0.36% (*w*/*v*) milk kefir grains powder and 0.5% (*w*/*v*) SCOBY-based membrane (both as freeze-dried culture), at 30 °C, for 5 days, in an aerobic stationary system, revealed an antifungal activity between 80 and 100% against *Aspergillus niger*, an antibacterial activity of 8–22 mm against *Escherichia coli* and *Bacillus* spp. And a titratable acidity of 445 °Th. The chemical composition of the obtained product had a positive impact on the functional properties of the fermented products in terms of the antimicrobial and antioxidant properties.

## 1. Introduction

The interest in the natural and multiple consortia of microorganisms such as milk/water kefir grains and SCOBY-based membranes has increased around the world because of a multitude of biochemicals for pharmaceutics and nutrition applications, taking into account their stability and metabolic potential complexity. Many microbial metabolites are bioactive molecules with antibacterial, anticancer, and antiviral activity [1] and several other therapeutic effects on the organism pathophysiology. These natural compounds demonstrated their superiority compared to synthetic compounds because they have the potential to be safe and nontoxic [2]. Usually, valuable metabolites are obtained from a single strain or a mixture of starter culture throughout controlled biotechnological conditions [3]. A high composition of biotics (pre-, post- and probiotics) can be obtained by fermentation with the natural and multiple consortia of the microorganisms from the artisanal cultures, such as milk, water kefir grains and kombucha membrane, these being microbiologically and biochemically stable, with a long time functionality, and able to work in a perfect synergism and symbiosis [4,5].

Bacteria, yeasts and bacteriophages encapsulated in a matrix of exopolysaccharides (EPS) (kefiran), are predominantly found in the microbiota of milk kefir grains. Cows’ milk is a typical substrate for the fermentation of milk kefir grains. Whey, colostrum, and other types of milk or milk derivatives are frequently used as fermentation substrates [6,7]. The bovine colostrum is rich in carbohydrates, proteins, lipids, growth factors (insulin-like growth factors—IGF I, IGF II), enzymes (antioxidant enzymes, proteinase, lipase and esterase), enzyme inhibitors (cysteine and trypsin inhibitors), nucleotides and nucleosides (nucleoside-5′-, di- and tri-phosphates, pyrimidine nucleosides), cytokines (IL-1β, IL-6, TNF-α, INF-γ, IL-1ra), fats (oleic and linoleic acids), vitamins (A, E, D, K, B, C) and minerals (H+, K+, Na+, Mg2+) [8]. Colostrum contains more proteins than the mature milk does, having a higher concentration of immunoglobulins. Significant levels of some antibacterial and antiviral substances such as lactoferrin, lactoperoxidase, and lysozyme are also found [9]. 

A wide variety of biochemicals with anti-tumor, anti-pathogenic, and immunological modulating properties can be produced by milk kefir grains due to their valuable fermentation properties [6]. Beneficial postbiotics derived from the colostrum as the fermentation substrate are produced through the metabolic activity of the microbial consortia [10]. 

SCOBY-based membranes comprise a complex microbiota of acetic and lactic acid bacteria and yeasts that typically ferment a specific medium based on black tea infusion supplemented with sugar [11]. Additionally, the biologic properties of the kombucha beverage are supported by the production of phenolic compounds and organic acids as a result of the microbiota metabolic activity in correlation with the rich in polyphenols phytochemical composition of black tea [12,13]. Due to its useful functional properties of fermented products, the kombucha membrane has gained popularity and its use as a starter culture has been extended in the fermentation of unconventional substrates, taking into account the potential of multiple microbiota to enhance the bioactive composition of fermented products with functional properties [14,15]. 

The use of multiple starter cultures cann the fermentation process, in regard to the diversification of the substrate composition and optimization of the main important biotechnological parameters, offers approachable perspectives to obtain fermented products that contain a large spectrum of biotics, with enhanced functional properties. The idea to combine kombucha membrane and milk kefir grains as a starter is innovative, and has been studied and exploited to a limited degree.

In this context, the aim of the study was the optimization of the fermentation process of a newly formulated medium based on black tea infusion, supplemented with bovine colostrum and sugar using the freeze-dried SCOBY-based membranes and milk kefir grains as starter cultures. The experiments were designed and the results were statistically analyzed based on mathematical modelling by using the Plackett–Burmann (PB) and response surface methodology (RSM) analysis. The influence of the selected independent variables with an impact on the process was analyzed, taking into account the responses correlated to the functional properties of the fermented product, i.e., bioactive content (organic acids, polyphenol and flavonoids), antimicrobial and antioxidant activities.

## 2. Materials and Methods

### 2.1. Milk Kefir Grains and Kombucha Membranes as Starter Cultures

Milk kefir grains, provided by a Romanian manufacturer, were firstly inoculated into pasteurized cows’ milk (3.5% fat) and incubated at 25 °C, for 48 h (Binder BF4000, Tuttlingen, Germany), and then transferred every 48 h in fresh milk, five times for the propagation [16]. Then, the fresh milk kefir grains were separated, washed with ultrapure water, supplemented with sterile inulin solution 10% (*w*/*v*) and freeze-dried at −80 °C using the Christ Alpha 1–4 LO plus equipment, Germany. The freeze-dried grains were grinded and stored at 4 °C. 

The kombucha membranes, purchased from a Moldavian supplier, were propagated by successive cultivation in a black tea infusion (3%) (Aaro Forstman Oy, Vantaa, Finland) supplemented with 7.5% (*w*/*v*) sugar and incubated at room temperature (22 ± 2 °C) for 10 days. Further, 100 g of fresh SCOBY-based membranes, cut into small pieces, were homogenized with 20% sterile inulin powder and then freeze-dried, being afterwards grinded and stored at 4 °C. 

### 2.2. Co-Fermentation with Starter Cultures Derived from Kombucha Membranes and Milk Kefir Grains

The formulated fermentation medium was prepared, taking into account the experimental matrix design (%, *w*/*v*): 1–3 black tea leaves infusion, in tap water at 90 °C, 5 min, 1–5 bovine colostrum (Axyar, Gembloux, Belgium), 5–10 white sugar, pH 6.7, which was then sterilized at 105 °C for 10 min. After cooling, the medium was inoculated with 0.2–0.3% (*w*/*v*) freeze-dried cultures of milk kefir grains and kombucha membranes, and incubated for 5 to7 days, at 30 °C, under stationary and aerobically conditions, in Erlenmeyer vessels. The unfermented control sample was obtained under the similar conditions. The obtained fermented products (FPs) were analyzed as fresh samples without any other preservation.

### 2.3. The Fermented Products’ (FPs) Analysis

#### 2.3.1. pH and Titratable Acidity (TA)

The pH value was determined using a digital pH-meter (Mettler Toledo, FiveEasy F20, Greifensee, Switzerland). For the TA assay, 4 g of FPs were solubilized in distilled water, in a 50 mL volumetric flask, then 10 mL of the samples were titrated with NaOH 0.1 N, in the presence of phenolphthalein. The TA was expressed as Thörner degrees (°Th), according to Association of Official Analytical Chemists method (1990) [17] and calculated with Equation (1):

(1) $$\mathrm{Titratable}\;\mathrm{acidity},\;\left[{}^{\circ}\mathrm{Th}\right]=V_{\mathrm{NaOH}}\times \mathrm{Dilution}\;\mathrm{factor}$$

#### 2.3.2. Antifungal Properties of the FPs

The antifungal activity was assessed against the *Aspergillus niger* MIUG M5 strain (part of the Collection of Microorganisms with the acronym MIUG, of the Bioaliment Research Platform of Faculty of Food Science and Engineering of Dunărea de Jos University, Galati, Romania). The mold strain was incubated for 96 h at 25 °C, on slants with YGC (Yeast Glucose Chloramphenicol Agar) medium. The fungal spores were mixed with a 0.9% sterile saline solution and brought to the desired concentration (10^5^ spores/mL). Further, 1 mL of the FPs was dispersed into Petri dishes and mixed with 20 mL of Potato Dextrose Agar (PDA) medium (Oxoid, England). Then, 10 μL of the spore suspensions were inoculated in the center of the solidified medium and incubated at 25 °C, for 96 h. The control sample was performed under the same conditions, without the addition of FPs. After the incubation period, the diameter of the fungal colony was measured, and the inhibition ratio (IR) was calculated using the following Equation (2) [18]:

(2) $$\mathrm{IR}=\frac{A_{c}-A_{t\;}}{A_{c}}\times 100$$

where, IR represents the growth inhibition ratio, A_c_—the diameter of colony growth for the control sample, A_t_—the diameter of colony growth on the medium supplemented with FPs.

#### 2.3.3. Antibacterial Properties of the FPs

The strains of *Bacillus subtilis* MIUG B1 (also part of the MIUG Collection), *Escherichia coli* ATCC 25922, and *Staphylococcus aureus* ATCC 25923 were grown on Potato Count Agar (PCA) medium (Scharlau, Barcelona, Spain), respectively, Mueller II Hinton Broth (Biolab, Hungary) with the addition of Agar Bacteriological (AB) medium (Scharlau, Spain) for 24 h, at 37 °C. Further, one colony of each strain was transferred onto Nutrient Broth (for *B. subtilis*) and Muller Hinton Broth, respectively, (for *E. coli* and *S. aureus*) and incubated overnight, at 37 °C. The overnight cultures were brought to the optical density (λ 600 nm) of 0.3, approximately 2.4 × 10^8^ CFU/mL. Afterwards, 0.5 mL of bacterial suspension were inoculated in the Petri dishes with a specific medium and holes with 8 mm in diameter were made and filled with 100 µL FPs. Then, the plates were incubated at 37 °C, for 48 h. After the incubation period, the inhibition zone was measured (mm) [19,20]. 

#### 2.3.4. Antioxidant Properties of the FPs

The antioxidant assay was performed by the DPPH (2,2-diphenyl-1-picrylhydrazyl) radical scavenging activity method and expressed as μM TE/mL, using a Trolox (6-hydroxy-2,5,7,8- tetramethylchroman-2-carboxylic acid) calibration curve as the reference. Firstly, the fermented samples were subjected to an extraction on an ultrasound bath (MRC. Ltd., Holon, Israel) for 30 min, at 40 °C. Further, they were centrifuged for 15 min, at 7000 rpm, at 4 °C. The DPPH was daily prepared in HPLC methanol, ≥99.9% and stored under dark conditions [21,22].

### 2.4. The Design and Optimization of the Fermentation

#### 2.4.1. Plackett–Burman Design of the Experiments

The chosen independent variables for PB analysis were selected based on the nutritional necessities and growth conditions of the used starter cultures’ microbial consortia. The effects of the six variables were analyzed, as follows: concentration of the black tea leaves, colostrum and sugar, fermentation time, and the dimension of the freeze-dried inoculum derived from the kombucha membrane and milk kefir grains (Table 1). Finally, the fermentation conditions, according to the experimental design, were established. 

The Plackett–Burman experimental design involved 15 runs based on the variation of 6 factorial points, and 3 center points. The analyzed responses were: pH, titratable acidity, antioxidant activity and antimicrobial activity (against *Bacillus subtilis*, *Escherichia coli*, *Staphylococcus aureus* and *Aspergillus niger*).

#### 2.4.2. Response Surface Methodology

After the identification of the most significant variables with impact on the fermentation responses, the optimization of the bioprocess was achieved by applying the central composite design (CCD) and the RSM through five levels of variation (minimum value, central value, maximum value, and axial points) (Table 2). The models were statistically validated (the *p* < 0.05 value among the values of the 2-way interaction model was considered). 

For the fermentative medium formulation and the fermentation process, the other parameters were kept constant, i.e., concentration of sugar 7.5% (*w*/*v*), concentration of the freeze-dried kombucha membrane culture 5% (*w*/*v*), the temperature of fermentation 30 °C and 5 days as the fermentation time.

### 2.5. Organic Acids and Phenolic Compounds Analysis

#### 2.5.1. Organic Acids Assay 

The organic acids’ separation and quantification was achieved using an Agilent 1200 HPLC system (Agilent Technologies, Santa Clara, CA, USA) equipped with an autosampler, degasser, quaternary pump, UV-VIS/FLD detectors and a thermostat. A Hamilton RPR X300 chromatographic column (250 × 4.1 mm, 7 µm particle size, Hamilton, Bonaduz, Switzerland) was used to elute the organic acids by a gradient with two mobile phases, A—KH_2_PO_4_, 20 mM, pH 2.4 and B—acetonitrile 90% (*v*/*v*) [23] as follows: min 0–80% A, min 10–40% A, min 12.5–40% A, min 12.6–80% A. The separation of the organic acids was performed using an injection volume of 20 µL, at a wavelength of 210 nm, 30 °C, and a mobile phase flow of 1.5 mL/min [24]. After 15 min of running, the data acquisition was performed using the Chemstation program, B.04.03 version. The identification of organic acids was performed by comparing the retention times of the targeted compounds to those obtained for the corresponding standards (Sigma–Aldrich, Taufkirchen, Germany). Their quantification was performed using calibration curves.

#### 2.5.2. Phenolic Compounds Assessment

The separation of polyphenols and flavonoids was performed using an Agilent 1200 HPLC system. A Synergi Max-RP-80 Å (250 × 4.6 mm, 4 µm particle size, Phenomenex, Torrance, California, USA) chromatographic column in a concentration gradient with ultrapure water: acetonitrile: formic acid = 87:3:10 as the mobile phase A—and mobile phase B—ultrapure water: acetonitrile: formic acid = 40:50:10. The gradient concentration was as follows: min 0–94% A, min 20–80% A, min 35–60% A, min 40–40% A, min 45–10% A. An injection volume of 20 µL, at the wavelength of 280 nm, respectively, 320 nm, at 30 °C and a mobile phase flow of 0.5 mL/min were used. After 80 min of running the method, the data acquisition was achieved using the B.04.03 version of the Chemstation software (Santa Clara, CA, USA). The identification of the compounds was performed by comparing their retention times to those obtained for the flavonoids and polyphenols standards (Sigma–Aldrich, Germany), under similar separation conditions. The quantification of the compounds from the samples was performed using the calibration curves based on the peak area [18,25].

### 2.6. Statistical Analysis

For the design of the experiments, the statistical analysis and the mathematical modelling associated with the PB and RSM analysis, Minitab 17 Statistical Software (v. 1.0, LLC, Pennsylvania State University, USA) was used. The statistical differences between the control and the optimized sample were evaluated based on the analysis of variance (ANOVA) and Tukey test, considering the confidence interval of 95% (*p* < 0.05). The provided results are the mean values obtained from three replicates followed by their corresponding standard deviations.

## 3. Results and Discussions

### 3.1. Selection of the Most Important Fermentation Parameters by PB Analysis

The biotechnological parameters such as the concentration and type of sugar and tea, the infusion time for the tea leaves, the time and temperature of the fermentation are important for the fermentation in kombucha production [26]. The microbial consortium from the kombucha membrane microbiota requires tea, sugar and around 7–14 days for fermentation, while the microbiota of the milk kefir grains ferment milk or milk derivatives after 2 to 4 days. The temperature of the fermentation can be varied between 20–30 °C, taking into account the optimal temperatures for the acetic acid bacteria and yeast (25–30 °C), and for the lactic acid bacteria (20–40 °C) [27]. 

The design associated with the PB analysis indicated fifteen experimental variants by varying the black tea concentration between 1–3% (*w*/*v*), the sugar concentration between 5–10% (*w*/*v*), the colostrum concentration 1–5% (*w*/*v*), 4 to 7 days of fermentation, the concentration of freeze-dried kombucha culture between 0.1–0.3% (*w*/*v*) and the concentration of freeze-dried milk kefir grains culture between 0.1–0.3% (*w*/*v*) (Table 3). 

The pH, the titratable acidity, the antibacterial activity against *B. subtilis*, the antifungal activity against *Aspergillus niger* and the antioxidant activity were the analyzed responses. 

The model was statistically validated based on the regression coefficients greater than 80% and the *p*-value (*p* < 0.05) in the case of pH. The most important parameters for the pH variation are: the fermentation time, the colostrum concentration and the concentration of the freeze-dried milk kefir grains starter culture. 

**Figure 1:**
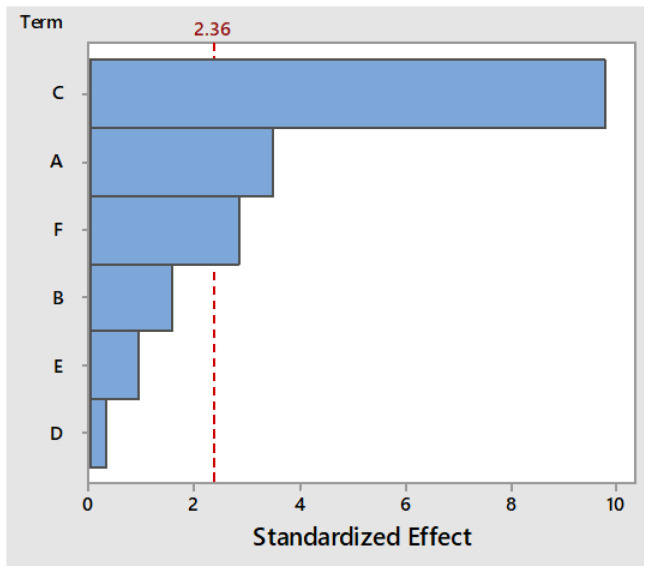
The Pareto chart of the effect of the studied independent variables upon the acidity of the fermented products.

For the titratable acidity, the most important parameters are: the black tea concentration (A), the colostrum concentration (C) and the freeze-dried milk kefir grains starter culture concentration (F). From the Pareto chart (Figure 1), it can be observed that the bars of the mentioned independent variables crossed the reference line at 2.36, which means that these parameters were statistically significant at the 0.05 level.

Additionally, the ANOVA analysis (Table 4) was performed for the analysis of variance and it was observed that the *p*-value for the most important factors mentioned in the Pareto chart were less than the significance level of 0.05 (which means that the factors were statistically significant in regard to the TA).

For the antibacterial activity, the mathematical model was statistically validated, the most significant parameters with influence on this response being the colostrum concentration and the fermentation time (limit *p* ≤ 0.05). Additionally, for the antioxidant activity, the black tea concentration, the colostrum concentration and the kombucha concentration were the most significant parameters in the variation of this response. Nevertheless, no significant parameters were observed for the antifungal activity as a response. 

The range of values obtained for the 15 analyzed samples with the PB design were 3.50–3.84 for pH, 75–187.50 °Th for the titratable acidity, 2.06–2.42 μM TE/mL for the antioxidant activity, 10.03–14.53 mm inhibition zone for the antibacterial activity and 82.17–86.89% of inhibition for the antifungal activity. 

During the kombucha fermentation, the pH values decreased from 5.34 to 2.53, after 14 days of fermentation, together with the increase in the fermentation time and acetic acid content. Recent studies, regarding the kombucha fermentation, highlighted a statistically significant correlation between the pH vs. time and acidity. Furthermore, the significant correlation was found for the acidity vs. the time of fermentation, saccharose content and pH [28]. Comparable values of the pH between 3.50–4.25 were detected for the milk substrate fermented with the milk kefir grains [29].

### 3.2. Optimization of the Fermentation Process by Response Surface Methodology (RSM)

Taking into account the previous results obtained by PB analysis, for the RSM, three significant parameters were selected, based on their statistical relevance (*p* < 0.05), and varied, as follows: between 1–5% for the concentration of bovine colostrum (A), between 1–3% concentration of black tea (B) and 0.1–0.3% concentration of milk kefir grains starter culture (F), while maintaining constant the other parameters that regarded the fermentative medium formulation, inoculum and fermentation temperature and time. A number of 20 runs were performed (Table 5). The analyzed responses were; the pH, titratable activity, antioxidant activity, antibacterial activity against *E. coli*, *S. aureus*, *B. subtilis* and antifungal activity against *A. niger*. 

**Figure 2:**
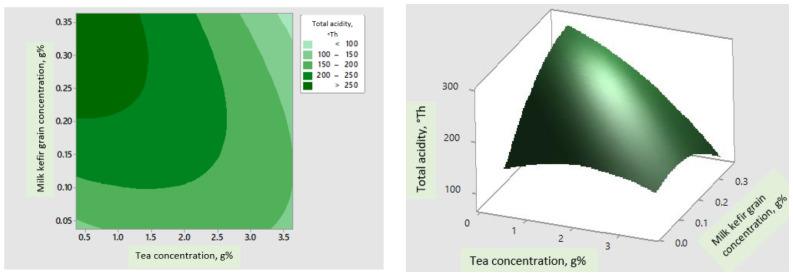
Contour (**left**) and surface plots (**right**) correlation effects between the milk kefir grains starter culture’s concentration and the tea concentration upon the TA.

Figure 2 presents the contour and surface plots of the interactions between the response, TA and two independent variables, the milk kefir grains starter culture’s concentration and the black tea concentration. The acidification potential increased with the increase in the milk kefir grains’ concentration and the decrease in the black tea concentration.

For all the 20 analyzed RSM samples, the range of values for the responses were 3.38–3.89 for pH, 100–344.8 °Th, 0.91–2.72 μM TE/mL for the antioxidant activity, 0–19 mm inhibition zone for the antibacterial activity and 90.41–100% of inhibition for the antifungal activity.

Based on these results, an optimized fermented product with high biologic potential was proposed, by fermentation of a medium, based of 6.27% (*w*/*v*) bovine colostrum powder, 1.64% (*w*/*v*) black tea, 7.5% (*w*/*v*) sugar, inoculated with 0.36% (*w*/*v*) freeze-dried milk kefir grains starter culture and 0.5% (*w*/*v*) freeze-dried kombucha membrane starter culture by an incubation under aerobic conditions, in a stationary system at 30 °C, for 5 days.

The results of the validated model were presented in Table 6. The experimental values were closer to the predicted ones, with a 95% composite desirability (D), which means that the individual and the correlated effects of the studied parameters are validated. 

The optimized fermented product was characterized by a high value of TA, an excellent antibacterial activity against pathogens and antioxidant properties which predict their functional properties. Instead, the control (unfermented sample) of the optimized product (based on 6.27% (*w*/*v*) bovine colostrum powder, 1.64% (*w*/*v*) black tea and 7.5% (*w*/*v*) sugar) showed lower values for the TA (12.5 ± 0.05 °Th) and for the antioxidant activity (2.105 ± 0.002 μM TE/mL) with no antimicrobial activity being detected. This data demonstrated that the optimization process with the co-culture of wild consortiums enhanced the bioactive properties of the fermented product.

For the antibacterial activity of the kombucha fermented product, the results are in accordance to recent studies where values of 14.5–19 mm inhibition zone against *S. aureus*, *E. coli*, and *B. cereus* were measured [30]. Moreover, the complete inhibition of *Bacillus cereus* and the partial inhibition for *Staphylococcus aureus* and *Escherichia coli* were measured for the milk product fermented with kefir grains [31].

### 3.3. The Organic Acids and Phenolic Content of the Fermented Product

The organic acids and the polyphenols’ concentrations, compounds formed in the fermented product obtained under optimized fermentation conditions, were assessed compared to the unfermented medium, with the same composition (control sample).

#### 3.3.1. Organic Acids’ Content

The HPLC analysis of the fermented product, obtained under the proposed optimized fermentation conditions, revealed five organic acids: lactic, citric, acetic, butyric and isovaleric (Table 7). An increasing concentration of the lactic and acetic acids could be observed from 7.77 mg/mL to 24.39 mg/mL, respectively, 9.14 mg/mL to 25.21 mg/mL, during the fermentation. Citric acid was also detected (5.77 mg/mL), although it could not be detected in the control sample (unfermented medium). Isovaleric acid remained constant at the level of 4.36 mg/mL. Butyric acid exhibited a small decrease during fermentation, from 81.63 mg/mL to 67.33 mg/mL. Compared to the control sample, in the fermented product, lactic and acetic acids increased up to three times while the citric acid increased up to 6 mg/mL. 

Usually, the main postbiotics found in kombucha are acetic acid, ethanol, and gluconic acid. The best substrates for the kombucha culture to create acetic acid were found to be green tea and black tea [27]. Other compounds include lactic acid, oxalic acid, saccharic acid, keto-gluconic acids, fructose, glucose, ethyl-gluconate, and tea components (catechins, theaflavins, flavonols, etc.). Many of these compounds have in vitro and in vivo functional properties. Lactic and acetic acids are known for their antimicrobial effects [32,33]. L-lactic acid, a biologically relevant isomer, promotes blood circulation and prevents constipation and gut rot. Additionally, it regulates the acid–alkaline balance and promotes the detoxification and the release of stomach enzymes [26]. Acetic acid exerts the positive effect on the yeast metabolisms to produce ethanol, which is then used by the acetic acid bacteria to multiply and to ferment. The ethanol and acetic acid have bioprotective effects against the microbial contamination of the fermented product and also for the safety assurance [28]. 

Other studies demonstrated that the tea’s effectiveness in showing the cytotoxicity effects increased after the fermentation (compared to the unfermented substrate). The fermented products also can lower the blood sugar levels. The tea tannins can also be transformed into pyrogallol groups, which have antibacterial properties. Additionally, the prostate cancer cells can be reduced by a lyophilized tea extract [29,30].

Our study demonstrated enhanced fermentation behavior in the production of lactic and acetic acids of the microorganisms from both artisanal starter cultures, in the fermentation of unconventional substrate (bovine colostrum, black tea and sugar). This composition of the fermented product was strongly correlated to their antimicrobial activity, both acids being known as antimicrobials.

#### 3.3.2. Polyphenols and Flavonoids Content

Catechin, epicatechin, epicatechin gallate, epigallocatechin, epigallocatechin gallate, gallocatechin, quercetin, kaempferol, myricetine and their glycosides are the most important tea polyphenols. These compounds can be transformed by microorganisms through their metabolic activity into catechins, gallic acid complexes (teaflavine, teaflavinic acids, thearubigine, theasinensis) and proanthocyanidin polymers by oxidation reactions [34]. The methylxanthines as caffeine, theophylline, and theobromine are present in a smaller concentration, around 2–4%. Volatile compounds are usually represented by terpenoids and amino acids degradation products, carotenoids, and linoleic acid. Tea also contains carbohydrates, vitamins (E, K, A, B), potassium, manganese and fluorine ions [35,36,37].

The main phenolic compounds detected in the analyzed fermented product were epicatechin 1062.69 µg/mL and caffeic acid 314.86 µg/mL, followed by quercetin 18.20 µg/mL, isorhamnetin 2.97 µg/mL and apigenin 0.22 µg/mL (Table 8). During the fermentation, the bioconversion of epicatechin into epicatechin gallate, epigallocatechin, and epigallocatechin gallate could be observed. As such, it was demonstrated that the acid-sensitive microbial cells released the catechins from the black tea, which could enhance the amount of polyphenols in the fermented tea [38]. The caffeine content of the black tea fermented with kombucha decreased from 217.81 µg mL/L to 100.72 µg mL/L [39]. 

Black tea leaves contain 0.24–0.52, 1.04–3.03, and 1.72–2.31 g/kg of myricetin, quercetin, and kaempferol, respectively, in terms of flavonol content on a dry weight basis [40]. The presence of chlorogenic acid, p-coumaric acid and kaempferol was also detected at 280 nm before the fermentation although after the fermentation, their content was not detectable mainly due to the metabolic activity of microorganisms from the microbiota of the milk kefir grains. Cardoso et al. (2020) detected 127 phenolic compounds in a fermented green and black tea with kombucha membranes. Among these, a 70.2% was represented by flavonoids (especially gallic, chlorogenic, syringic, and protocatechuic acids, rutin and vitexin), 8.4% by polyphenols, 18.3% by phenolic acids and other compounds [39]. 

Due to their ability to rapidly enter cells, some flavonoids can reach the nucleus, and enhance the interactions between the cytosolic and nuclear molecules in cultured neurons. For example, a concentration between 25 and 50 μM of quercetin can help the neurons survive the oxidative stress caused by cytotoxic substances such as glutamate, amyloid peptide, and H_2_O_2_ [2]. 

The phenolic composition of the fermented product obtained under optimized fermentation conditions was correlated to the antioxidant activity thus predicting their functional properties.

## 4. Conclusions

The innovative approach of this study converged from the use of water kefir grains and kombucha membranes, as freeze-dried starter cultures, to achieve fermentation processes under unconventional conditions. By correlating the biotechnological parameters specific for each consortium (medium composition, inoculum type and concentration, temperature and fermentation time), design of the experiments, mathematical modelling and the statistical analysis proposed by PB and CCD analysis, several independent variables were established with an impact on the functional properties of the fermented product. 

The results demonstrated that the microorganisms within the microbiota of the studied artisanal cultures, were able to work together, in synergism and symbiosis, to enhance the fermented product’s functional characteristics. Under the tested conditions, the most important parameters to obtain a fermented product with increased functionality were established as the concentration of bovine colostrum, the concentration of black tea infusion and the concentration of the freeze-dried cultures.

An acidification potential of 456.25 °Th, an antioxidant activity of 2.42 μM TE/mL and an antibacterial activity of 8.0, 8.67 and 21.67 mm against *S. aureus*, *E. coli* and *B. subtilis*, respectively, were detected for the fermented product obtained under the optimized fermentation conditions. The fermented product contained postbiotics with a functional potential, such as organic acids (butyric—67.33 mg/mL, acetic—25.21 mg/mL, lactic—24.39 mg/mL, citric—5.77 mg/mL and isovaleric acids—4.36 mg/mL) and phenolic compounds (gallic and caffeic acids—7.40, respectively, 314.86 µg/mL, epicatechin—1062.69 µg/mL, quercetin—18.20 µg/mL, and isorhamnetin 2.97 µg/mL). This composition improved the antimicrobial and antioxidant activities and recommend the obtained product as a valuable bioingredient with several possible applications in the food and feed formulation, with impact on controlling the oxidation process, microbial spoilage and safety assurance.

## Figures and Tables

**Table 1:** Independent variables and their levels of variation established through the PB design.

Independent Variables	Minimum Value, −1	Maximum Value, +1
**A**, Concentration of black tea, % (*w*/*v*)	1.0	3.0
**B**, Concentration of sugar, % (*w*/*v*)	5.0	10
**C**, Concentration of colostrum, % (*w*/*v*)	1.0	5.0
**D**, Fermentation time, days	5.0	7.0
**E**, Concentration of the freeze-dried culture derived from SCOBY-based membranes, % (*w*/*v*)	0.2	0.3
**F**, Concentration of the freeze-dried culture derived from milk kefir grains, % (*w*/*v*)	0.2	0.3

**Table 2:** Independent variables and their levels of variation in the CCD for RSM.

Independent Variables	Levels of Variation				
	−1	0	+1	−α	+α
**A**, Concentration of black tea, % (*w*/*v*)	1.0	2.0	3.0	0.367	3.633
**C**, Concentration of colostrum, % (*w*/*v*)	1.0	3.0	5.0	−0.266	6.266
**F**, Concentration of milk kefir grain, %(*w*/*v*)	0.1	0.2	0.3	0.0367	0.3633

**Table 3:** Design of the experiments by PB analysis and the obtained responses according to the variation of the independent variables.

Sample	Independent Variables *						Responses				
	A	B	C	D	E	F	pH	Titratable Acidity, °Th	Antioxidant Activity, μM TE/mL	Antibacterial Activity against *B. subtilis*, mm	Antifungal Activity against *A. niger*, % RGI %
1	2.0	7.5	3.0	5.5	0.2	0.2	3.75	137.50	2.37	13.71	86.23
2	3.0	10.0	1.0	7.0	0.3	0.1	3.52	100.00	2.41	11.37	83.72
3	3.0	5.0	5.0	4.0	0.1	0.1	3.84	137.50	2.40	13.20	84.11
4	1.0	10.0	5.0	4.0	0.3	0.1	3.78	150.00	2.20	14.53	82.95
5	3.0	5.0	5.0	7.0	0.1	0.3	3.74	175.00	2.42	13.70	82.17
6	1.0	10.0	1.0	4.0	0.1	0.3	3.50	75.00	2.29	13.37	83.72
7	2.0	7.5	3.0	5.5	0.2	0.2	3.72	137.50	2.38	13.20	86.89
8	1.0	5.0	1.0	7.0	0.3	0.3	3.50	87.50	2.21	13.70	85.27
9	1.0	5.0	1.0	4.0	0.1	0.1	3.53	75.50	2.36	12.70	84.93
10	3.0	10.0	5.0	4.0	0.3	0.3	3.76	187.50	2.12	11.03	84.59
11	3.0	5.0	1.0	4.0	0.3	0.3	3.51	112.50	2.36	14.20	85.27
12	2.0	7.5	3.0	5.5	0.2	0.2	3.71	150.0	2.36	14.53	86.56
13	3.0	10.0	1.0	7.0	0.1	0.1	3.53	100.00	2.42	12.20	84.25
14	1.0	5.0	5.0	7.0	0.3	0.1	3.74	125.00	2.08	10.03	85.96
15	1.0	10.0	5.0	7.0	0.1	0.3	3.69	162.50	2.06	13.70	86.31

* A—Concentration of tea, % (*w*/*v*), B—Concentration of sugar, % (*w*/*v*), C—Concentration of colostrum, % (*w*/*v*), D—Fermentation time, days, E—Concentration of kombucha starter culture % (*w*/*v*), F—Concentration of milk kefir grains starter culture, % (*w*/*v*).

**Table 4:** ANOVA analysis of variance for the TA.

Source	DF	Adj SS	Adj MS	F-Value	*p*-Value
Model	7	16285.7	2326.5	17.86	0.001
Linear	6	15536.6	2589.4	19.87	0.000
Back tea concentration, %	1	1564.1	1564.1	12.00	0.010
Sugar concentration, %	1	320.3	320.3	2.46	0.161
Colostrum concentration, %	1	12480.7	12480.7	95.79	0.000
Time of fermentation, days	1	12.0	12.0	0.09	0.770
Kombucha starter culture concentration, %	1	114.1	114.1	0.88	0.381
Milk kefir grains starter culture concentration, %	1	1045.3	1045.3	8.02	0.025
Curvature	1	749.1	749.1	5.75	0.048
Error	7	912.1	130.3		
Lack-of-Fit	5	807.9	161.6	3.10	0.262

**Table 5:** CCD and the analyzed responses in correlation with the independent variables variation.

Run		Responses								
	A *	C	F	pH	Total activity, °Th	Antioxidant Activity, μM TE/mL	Antibacterial Activity against *E.coli*, mm	Antibacterial Activity against *S.aureus*, mm	Antibacterial Activity against *B.subtilis*, mm	Antifungal Activity against *A.niger*, %
1	1.00	1.00	0.30	3.55	225.00	1.30	2.17	0.00	10.50	93.73
2	2.00	3.00	0.20	3.71	229.37	2.01	4.00	0.14	10.05	92.95
3	2.00	3.00	0.20	3.80	220.00	2.12	1.83	0.00	8.55	93.15
4	3.00	1.00	0.30	3.71	100.00	2.47	0.00	0.00	4.83	100.00
5	1.00	5.00	0.30	3.49	344.80	1.94	6.50	3.83	18.50	100.00
6	1.00	5.00	0.10	3.79	212.62	1.31	2.67	3.52	14.50	93.38
7	3.00	5.00	0.30	3.76	250.00	2.44	3.50	0.00	10.33	100.00
8	1.00	1.00	0.10	3.38	229.00	1.35	2.50	0.00	11.75	90.76
9	2.00	3.00	0.20	3.72	234.20	2.01	4.00	0.00	11.12	92.98
10	3.00	5.00	0.10	3.86	187.50	2.35	0.00	0.00	8.17	90.41
11	2.00	3.00	0.20	3.82	215.60	2.07	2.66	0.00	7.95	94.23
12	3.00	1.00	0.10	3.65	193.75	2.72	2.00	0.00	7.83	100.00
13	2.00	3.00	0.20	3.76	243.75	2.14	2.50	0.00	9.55	93.68
14	3.63	3.00	0.20	3.80	150.00	2.41	0.00	0.00	6.67	100.00
15	2.00	6.27	0.20	3.71	308.20	2.08	5.50	3.20	16.00	100.00
16	2.00	−0.27	0.20	3.41	225.00	2.23	2.00	0.00	8.91	100.00
17	2.00	3.00	0.20	3.83	206.25	2.16	3.35	0.00	9.02	93.54
18	2.00	3.00	0.36	3.74	212.50	2.20	2.83	0.00	7.67	100.00
19	2.00	3.00	0.03	3.89	162.50	2.18	1.83	0.00	7.17	91.40
20	0.37	3.00	0.20	3.47	238.80	0.91	5.18	3.44	16.23	96.03

* A—Concentration of black tea (%, *w*/*v*), C—Concentration of bovine colostrum (%, *w*/*v*), F—Concentration of milk kefir grains starter culture (%, *w*/*v*).

**Table 6:** Model validation of the studied biotechnological parameters for the optimized fermented product.

Response	Predicted Value	Experimental Value
Titratable acidity, °Th	434.50	456.25 ± 0.16
Antioxidant activity, μM TE/ mL	2.45	2.42 ± 0.01
Antibacterial activity against *E. coli*, mm	9.50	8.67 ± 0.10
Antibacterial activity against *S. aureus*, mm	5.07	8.00 ± 0.22
Antibacterial activity against *B. subtilis*, mm	21.98	21.67 ± 0.10
Composite desirability	0.95	

**Table 7:** The organic acids’ concentrations after the medium fermentation based on bovine colostrum, black tea and sugar with milk kefir grains and freeze-dried kombucha membrane starter culture, under optimized conditions.

Organic Acids	Concentration, mg/mL	
	Control Sample	Fermented Product
Lactic acid	7.77 ± 2.14 ^b^	24.39 ± 0.04 ^a^
Citric acid	ND *	5.77 ± 0.01 ^a^
Acetic acid	9.14 ± 0.00 ^b^	25.21 ± 0.10 ^a^
Butyric acid	81.63 ± 0.07 ^a^	67.33 ± 0.05 ^b^
Isovaleric acid	4.36 ± 0.01 ^a^	4.36 ± 0.01 ^a^

Different letters in a row denote significant differences between the control and the fermented product (*p* < 0.05). * ND—Not Detectable.

**Table 8:** Phenolic acids and flavonoids’ content of the fermented product obtained under optimized biotechnological conditions.

Bioactive Compounds	Control Sample, µg/mL		Optimized Sample, µg/mL	
	280 nm	320 nm	280 nm	320 nm
Gallic acid	170.51 ± 5.73 ^a^	27.71 ± 0.08 ^A^	71.40 ± 4.82 ^b^	7.52 ± 2.34 ^B^
Epicatechin	ND	ND	1062.69 ± 53.50 ^a^	347.84 ± 50.81 ^A^
Caffeic acid	464.45 ± 49.00 ^a^	97.16 ± 1.44 ^A^	314.86 ± 28.10 ^b^	7.79 ± 2.14 ^B^
Chlorogenic acid	54.66 ± 8.11 ^a^	ND	ND	ND
p-Coumaric acid	28.89 ± 0.11 ^a^	ND	ND	ND
Quercetin	ND	ND	18.20 ± 0.02 ^a^	ND
Apigenin	ND	ND	0.22 ± 0.00 ^a^	ND
Isorhamnetin	4.60 ± 0.10 ^a^	ND	2.97 ± 0.03 ^b^	ND
Kaempferol	282.30 ± 28.09 ^a^	9.78 ± 1.00 ^A^	ND	ND

Different lowercase letters in a row denote significant differences between the control and the optimized sample at 280 nm, whereas the different uppercase letters in a row denote the significant differences between the control and the optimized sample at 320 nm (*p* < 0.05).

## Data Availability

Data is contained within the article.
